# Decentralized clinical trials are better for the participants and for the planet: the case study of a double-blind randomized controlled trial in Singapore (PROMOTE study)

**DOI:** 10.3389/fpubh.2024.1508166

**Published:** 2025-01-13

**Authors:** Lisa R. Fries, Nadia Khaled, Ivan Viveros Santos, Elvira Suniega-Tolentino, Motshewa Sesing, Melissa P. S. Toh, Chui Yuen Yang, Shiao Yng Chan, Sara Colombo Mottaz

**Affiliations:** ^1^Nestlé Institute of Health Sciences Nestlé Research, Société des Produits Nestlé SA, Beijing, China; ^2^Clinical Research Unit, Nestlé Research, Société des Produits Nestlé S.A., Lausanne, Switzerland; ^3^CIRAIG, Chemical Engineering Department, Polytechnique Montréal, Montreal, QC, Canada; ^4^Clinical Research Unit, Nestlé Research, Société des Produits Nestlé S.A., Singapore, Singapore; ^5^Singapore Institute for Clinical Sciences Agency for Science, Technology and Research, Singapore, Singapore; ^6^Department of Obstetrics and Gynaecology, Yong Loo Lin School of Medicine, National University of Singapore, Singapore, Singapore

**Keywords:** decentralized clinical trial, RCT, environmental impact, life-cycle assessment, maternal health, research participant experience

## Abstract

**Introduction:**

Novel technologies have enabled the decentralization of many aspects of clinical trials, but little research has been done on the impact of these changes on the participant experience, trial operations, or the environment.

**Methods:**

A fully decentralized clinical trial conducted in Singapore is used as a case study to evaluate the operational outcomes, environmental impact (via life cycle assessment), and participants experience (qualitative interviews) of the decentralized model compared to a traditional study with in-person visits.

**Results:**

The decentralized study achieved high participant retention rates (97%) and high completion rates for clinical data, even for biological samples. Participants found the decentralized model to be convenient and safe, especially during the pandemic. Moreover, the decentralized model was found to be more environmentally friendly and less detrimental to human health compared to traditional face-to-face clinical trials, primarily by reducing participants’ use of cars for site visits.

**Discussion:**

While this study focused on the environmental impact, it is important to consider other factors such as participant safety, convenience, and data quality when evaluating the suitability of a decentralized clinical trial approach. Careful planning of data flow, database structure, and data protection measures is essential. This study contributes to improving the environmental footprint of clinical trials. Environmental sustainability should be among the factors that are evaluated when selecting trial models. Decentralized and hybrid clinical trials offer efficiency, effectiveness, and environmental benefits. Further research and adoption of these approaches are encouraged.

## Introduction

The global trend to adopt digital technologies is also transforming clinical trial operations, potentially making clinical trials more efficient, cost-effective, and inclusive by increasing the possibility of remote interactions with study participants (1). Trial decentralization involves bringing study activities to the participant, rather than using the traditional paradigm of bringing participants to a central trial site, such as a hospital or university lab (2). The shift of clinical trial activities to be closer to participants has been enabled by a constellation of technologies and services. Tools such as electronic consent forms, tele-healthcare, mobile apps and sensors, remote participant monitoring, and electronic clinical outcome assessments (eCOAs) allow investigators to stay connected with trial participants without in-person visits. In addition to these digital tools, home visits from healthcare professionals, home delivery of study products, and collection of biological samples by courier bring study activities even closer to participants’ homes. Those tools allow for a broad spectrum of trial operations, from fully decentralized to different levels of hybrid models. While most clinical trials are not likely to be entirely decentralized, they can adopt one or more decentralization elements based on suitability for their end points, participant populations, and type of intervention.

The COVID-19 pandemic has significantly catalyzed the adoption of decentralized clinical trials and digital tools, since many health system resources were consumed by COVID-19-related care and participants’ ability to access traditional trial sites was limited by physical distancing and lockdown constraints (3). Many organizations turned to decentralized or hybrid study to mitigate the impact of the pandemic on research (4, 5), thus accelerating the uptake of this approach in recent years.

In this article, we focus on two important aspects linked with the decentralization of clinical trial activities: the participants’ experience and the environmental impact. Our study was a fully decentralized double-blind randomized controlled trial (RCT) recently run in Singapore. The PROMOTE trial was well suited to be conducted using a decentralized approach, as the primary outcomes (maternal mental health) could be measured by questionnaire and the intervention (probiotics) could be easily self-administered at home. Further, as we targeted a health-sensitive population (pregnant women), there could have been greater motivation to be socially distanced and avoid unnecessary travel and interactions, as would be required in a traditional clinical trial model. The availability of local infrastructure and services (e.g., internet connectivity, courier services) also contributed to making this a feasible option for this study.

## Methods

### Clinical trial design and operations

The PROMOTE RCT study^1^ aimed to assess the effects of a probiotic (*Bifidobacterium longum* NCC3001) on perinatal mood and stress (6). Participants were recruited during their third trimester of pregnancy (28–32 weeks gestation) and randomized into one of three study arms (probiotic for whole study period, probiotic only in postnatal phase, and placebo control) and followed until 3 months postpartum. Women were eligible to participate if they were at least 21 years old, pregnant with a singleton pregnancy, able to answer questionnaires in English, and scored at least a 5 on either the anxiety or depression subscale of the Hospital Anxiety and Depression Scale [HADS; (7)]. Exclusion criteria included unwillingness to follow study procedures, a diagnosed food allergy, and having taken probiotic supplements in the past 4 weeks before screening or pharmacological treatment for anxiety or depression in the 12 weeks before screening.

To mitigate the risk of unpredictable conditions of executing a clinical trial during the pandemic, the end-to-end clinical operations of this trial were fully decentralized without any in-person visits requiring participants to travel to a study site. All study “visits” were conducted from the comfort of the participant’s home virtually via video chat. Questionnaires were completed digitally using a mobile app called iMedidata. Stress measures based on heartrate variability were also collected using an app called Anura. Verbal instructions for download and use of the apps used for data collection were given during the remote visits. Intervention products and sample collection materials were sent to the participants and biological samples (saliva and stool) were returned by courier. The study was approved by the A*STAR (The Agency for Science, Technology and Research) Institutional Research Board (reference number 2020-065). All participants provided written informed consent.

A community recruitment approach was used to reach prospective participants, including (1) social media advertising; (2) online parenting websites, forums, platforms, and group chats; (3) physical posters on public transport; (4) brochures in mailboxes and partner locations; and (5) word-of-mouth. Most participants reported that they first heard about the study through advertisements on social media platforms, primarily Facebook and Instagram, where advertisements targeting users aged 21–40 years old had run for approximately 42 weeks until recruitment targets were met. In total 520 women registered their interest online, 205 were enrolled, and of these, 184 were randomized into the trial.

The investigational product adherence rate was defined as product consumed on at least 80% of study days, as reported by participants. Protocol deviations were monitored by the study team to ensure the completeness, accuracy, and reliability of the collected study data. The full study design has been published in Toh et al. (6).

### Qualitative assessment of participant experience

There have been relatively few studies of participant subjective experience in RCTs in general (8). This qualitative study used semi-structured interviews by video chat to understand the thoughts and opinions of a subset of participants (*n* = 18) who completed the clinical trial. The last group of participants from the clinical trial (*n* = 82) were asked during their last study visit if they would consent to be contacted for future research, of which 68 (83%) agreed. Of these, 34 (50%) were selected and contacted for the qualitative study about 6–9 months after the end of their participation in the trial. The selection aimed to include a diverse group of participants, based on their age, baseline scores on the Hospital Anxiety and Depression Scale (HADS; (7)), ethnicity, and education level. Of those contacted, 21 (62%) agreed to be interviewed. Interviews were conducted in random order by a single researcher for consistency. After 18 interviews, a saturation in themes was reached and a decision was taken to stop conducting further interviews. The characteristics of the participants who were interviewed is summarized in Table 1. The ages ranged from 21 to 39 years (mean 33 years; SD 4.5), with most participants being primiparous (72%) and having completed at least a university degree (89%). Baseline HADS scores ranged from 12–22 (mean 17; SD 2.5). Just over half (56%) of participants were of Chinese descent (the predominant ethnicity participating in the trial) with the remaining 44% comprising five other ethnicities. The distribution of participant characteristics can be found in Table 1.

**Table 1:** Characteristics of the subset of participants who participated in the qualitative follow-up study.

Participant characteristics	Category	Number of participants
Age	21–25	1
	26–30	3
	31–35	8
	36–40	6
	>20	3
Hospital Anxiety and Depression Scale (HADS) score at screening	<13	1
	14–15	5
	16–17	4
	18–19	5
	>20	3
Ethnicity	Chinese	10
	Malay	3
	Indian	2
	Korean	1
	Caucasian	1
	Mixed Asian	1

Four main topics were covered in the interview guide: (1) participants’ understanding of the purpose of the clinical trial, their perception and overall experience; (2) what convinced the participants to enroll in the clinical trial; (3) how they felt about the tasks involved in their participation in the clinical trial; and (4) preferences between remote and in person clinical trials. Interviews were transcribed by the interviewer and then an inductive thematic analysis was performed by another member of the research team (8).

### Environmental impact of the clinical operations

We aimed to assess the environmental impact of our fully decentralized trial, compared to an equivalent study with entirely center-based operations (the traditional model). We employed a *Life Cycle Assessment (LCA)* (9, 10) approach, which models all processes associated with carrying out a clinical trial, no matter when and where they take place. It subdivides the life cycle into elementary processes and develops a *Life Cycle Inventory (LCI)* of all associated inputs (i.e., natural resources) and outputs (i.e., emissions to air, water, and soil). These inputs and outputs are further converted into environmental impact scores using life cycle impact assessment methods. Accordingly, our LCA study intended to identify the principal contributors to environmental impact scores, to ultimately support environmentally conscious decision-making in clinical trial design. Specifically, the study compared the environmental impact of one subject (i.e., functional unit) participating in the two clinical trial models (decentralized vs. traditional).

**Figure 1:**
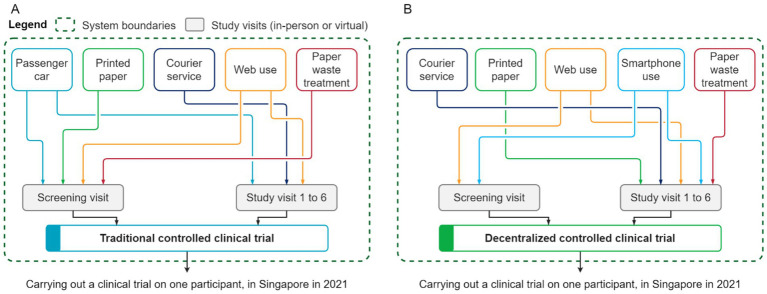
Simplified system boundaries of the analyzed operational models for conducting a clinical trial: center-based/traditional **(A)** and decentralized **(B)**.

The scope of this LCA study is shown in Figure 1. It comprises end-to-end system boundaries for the two clinical trial operational models. For this study, we assumed that the outcome, the rates of participant withdrawal and the analyzed systems of the decentralized clinical trial would be equivalent to the traditional trial. The system boundaries comprised a screening visit and six clinical visits (whether in-person or virtual), which included the transportation of the participant to the study center (traditional model), the use of printed paper for questionnaires and booklets, paper waste management, internet access, and the use of a smartphone (decentralized model). In addition, a courier service was included to model the single delivery of all sampling materials at the start of the trial and the collection back of biological samples at relevant time-points; however, the actual biological sampling kit is excluded from the system boundaries because it is the same in both clinical trial models (Figure 1).

As aforementioned, a *Life cycle inventory (LCI)* itemizes and quantifies all natural resources used and all the emissions of pollutants to water, air, and soil across all elementary processes associated with the life cycle of a clinical trial. It uses two types of data: primary data that describes the system under study; complemented by secondary data that describes the system environmentally with emission factors obtained from LCI databases. The primary data was built from protocol design and clinical operational considerations. It includes the characteristics of the analyzed operational models for conducting clinical trials, particularly the number of visits (whether in-person or virtual), the use of paper and internet access to respond to questionnaires, the transport of the participant to the study center (traditional model), courier services for delivering collection kits and collecting biological samples (mainly for the decentralized model, and to some extent in the traditional model, if the participant was unable to provide fresh stools on the day of the study visit), and paper waste treatment (Figure 1).

**Figure 2:**
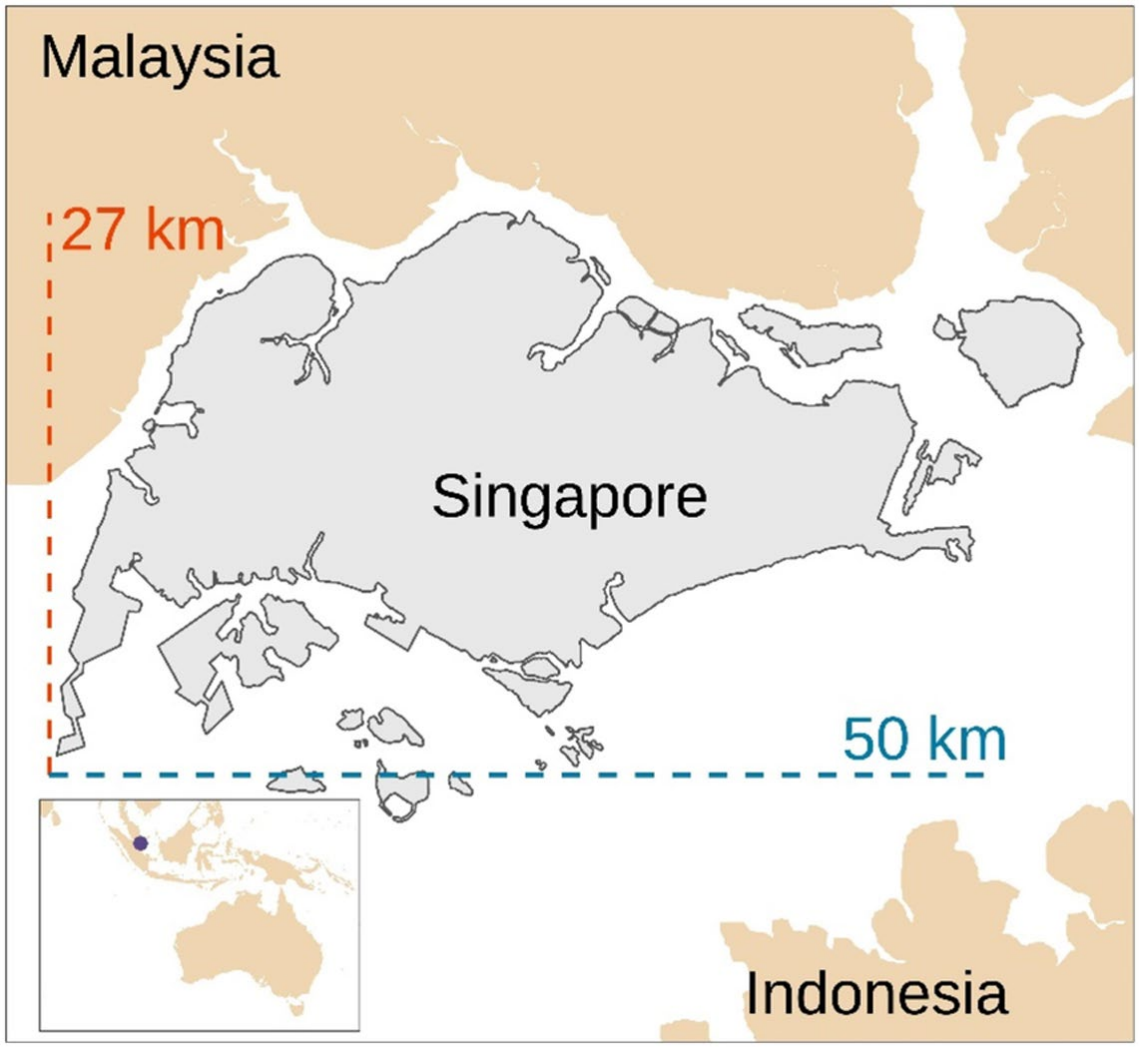
Map of Singapore. Our representation based on a shapefile from Hijmans (29).

Table 2 describes the main primary data of the assessed models. Given the inherent variability of the data, average values were considered for modeling the systems. For instance, the virtual visits ranged in duration between 15 and 30 min, therefore, a mean value of 22.5 min was considered for modeling the internet access, smartphone and computer use, and electricity consumption related to this operation. Furthermore, participants were enrolled from across Singapore. Considering that the mainland of Singapore measures approximately 27 km from north to south and 50 km from east to west (Figure 2), an average distance of 25 km was considered between the study center, the subject’s location, and the courier service base to model the transport of the participant to the study center and courier services (from the subject’s location to courier service base and from study center to courier service base). It was assumed that participants would have preferred traveling by car (especially when heavily pregnant) rather than using public transport.

**Table 2:** Main characteristics of analyzed models for conducting a clinical trial.

Input/Controlled clinical trial	Traditional	Decentralized
Phone call duration	N/A	22.5 min. (15–30 min.) per virtual visit
Internet use duration	5 min./page of data entry of questionnaires Total 144 pages	5 min./page of questionnaire (participant) 45 pages at screening (12 pages for consent form, 3 pages of eligibility screening including HADS, 30 pages for screening visit questionnaires) 111 pages for questionnaires during V1-V6
Paper	4.5 g/page	
Consent form at screening visit	24 pages	N/A
Demographics questionnaire at screening	7 pages	N/A
Eligibility screening, including HADS	3 pages	N/A
Questionnaires (visit 1 to 6)	Total pages across all visits: 111	N/A
Screening visit (questionnaire)	10 pages	N/A
Instruction booklet for sample collection	8 pages	8 pages
Transport	25 km/displacement	25 km/displacement
Screening visit	100% by car/taxi (1 visit: 2 displacements for a return trip)	N/A
Study visits	100% by car/taxi (6 visits: 12 displacements)	N/A
Courier service (light commercial vehicle; quantified by mass *displacement distance)	4 displacements with 1 kg mass of 25 km and 8 displacements empty handed. (Assuming the participant is unable to provide fresh stool on the day of the visit, the courier service is required for 4 stool collections.)	Item (mass) (Number of displacements): Sampling kit delivery (5 kg) (single delivery: 1 displacement with mass, 2 displacements empty handed) IP Study product supply and return (1 kg) (1 supply, 1 combined supply & return, 1 return only: 3 displacements with IP, 4 displacements empty handed) Biological sample (1 kg) pick-up for saliva and fresh stool separately (9 displacements with mass, 18 displacements empty handed)

^*^Each courier trip required 2–3 “displacements,” as the courier needed to travel from base to and from the participant’s home and in some cases, to collect materials from or drop samples at the study site en route.

The primary data was complemented with secondary data from the ecoinvent v3.8 LCI database. The LCI secondary data was geographically contextualized to Singapore, particularly the electricity grid mix and waste management activities, such as waste recycling rates. The analyzed systems for conducting clinical trials were modeled in SigmaPro v9.3.0.3 software, according to the systems illustrated in Figure 1.

#### Life cycle impact assessment (LCIA) and sensitivity analyses

The IMPACT World+ (2.0 V2.00) method (11) was employed to compute the life cycle impact assessment (LCIA) of the clinical trial. IMPACT World+ incorporates several recent developments in LCIA, namely spatial differentiation, and it is a modern version of the older IMPACT 2002+, LUCAS, and EDIP methods. In addition, IMPACT World+ computes endpoint results from elementary flows (emissions and primary resources) according to cause-effect chains, instead of employing factors to translate midpoint indicators, which is the current approach employed by other LCIA methods (11).

The environmental performance of the two analyzed models for conducting a clinical trial was evaluated based on one midpoint and two endpoint indicators from IMPACT World+. The *climate change in the short-term* midpoint indicator (during the first 100 years after the emission) was selected for reporting the carbon footprint of clinical trials (kg CO_2_eq/clinical trial). The two endpoint indicators of the impacts on *human health (HH)* and *ecosystem quality (EQ)* areas of protection (AoP) were computed. Consequently, the use of these three indicators facilitated the assessment of the clinical trial models in relation to the three primary dimensions of the triple planetary crisis: climate change, human health impacts from pollution, and biodiversity loss (12).

The impacts on human health are assessed in disability-adjusted life years (DALY). DALYs represent the overall burden of disease by combining years of life lost due to premature mortality and years lived with disability because of pollution. The DALY calculation applies a damage factor to disease cases, reflecting the severity of the disease in terms of both mortality and morbidity. This metric enables a comprehensive assessment of human health impacts within the framework of LCA, considering both disease incidence and its long-term effects on quality of life (13). The human health endpoint encompasses human toxicity non-cancer, human toxicity cancer, water availability, particulate matter formation, photochemical oxidant formation, ozone layer depletion, and ionizing radiation (11). The impacts on ecosystem quality are evaluated in terms of the potentially disappeared fraction of species over an area of one square meter over one year (PDF·m^2^·yr). The ecosystem quality endpoint comprises freshwater acidification, land transformation and land occupation, marine acidification, marine eutrophication, freshwater ecotoxicity, freshwater eutrophication, ionizing radiation, terrestrial acidification, and water availability (11). To avoid double counting of impact, the specific contribution of climate change to human health and ecosystem was omitted.

Two sensitivity analyses were carried out to assess the robustness of the conclusions on the environmental performance of the models and to analyze the influence of some key parameters on the LCIA results. The first sensitivity analysis involved the calculation of the LCIA with an alternative impact method. For this purpose, the LCIA of clinical trials were computed with the ReCiPe (H) method (14). ReCiPe (H) is a widely used LCA method that characterizes environmental impacts across multiple categories, including climate change, human health, and resource depletion. It is designed to provide both midpoint and endpoint results, offering a comprehensive understanding of environmental effects (14). In comparison to the IMPACT World+ method, ReCiPe (H) provides similar geographical coverage and impact category breadth. However, ReCiPe (H) is updated less frequently, with the most recent version dating to 2016, whereas IMPACT World+ includes more up-to-date data. Additionally, ReCiPe (H) includes 3,164 elementary flows, compared to 3,438 in IMPACT World+, making its coverage of elementary flows slightly more limited. The decision to use ReCiPe (H) in the sensitivity analysis was driven by the need to assess how an alternative impact assessment method might influence the environmental performance results of the clinical trial models and to enable comparison with similar studies in future research. The second sensitivity analysis corresponded to evaluating the influence of the distance of transport between the participant’s home and the study center and between the courier base and the participant’s home.

### Scenario for the traditional clinical trial including public transport

In addition to the reference scenario of the traditional clinical trial, as described in Table 2, one additional scenario was developed in order to assess the influence of including public transport for participant travel. Since the PROMOTE study was conducted during the COVID-19 pandemic, the base scenario for the traditional model only included transport by passenger car (private car or taxi). However, a second scenario was designed to evaluate the environmental profile of conducting the clinical trials in a post-pandemic situation, in which some participants would use public transportation. In this scenario, we assumed that 50% of participants would travel to the study site by car, 25% by bus, and 25% by Mass Rapid Transit (MRT) Metro, based on typical transportation patterns of Singaporean residents (15).

## Results

### Operational outcomes

**Figure 3:**
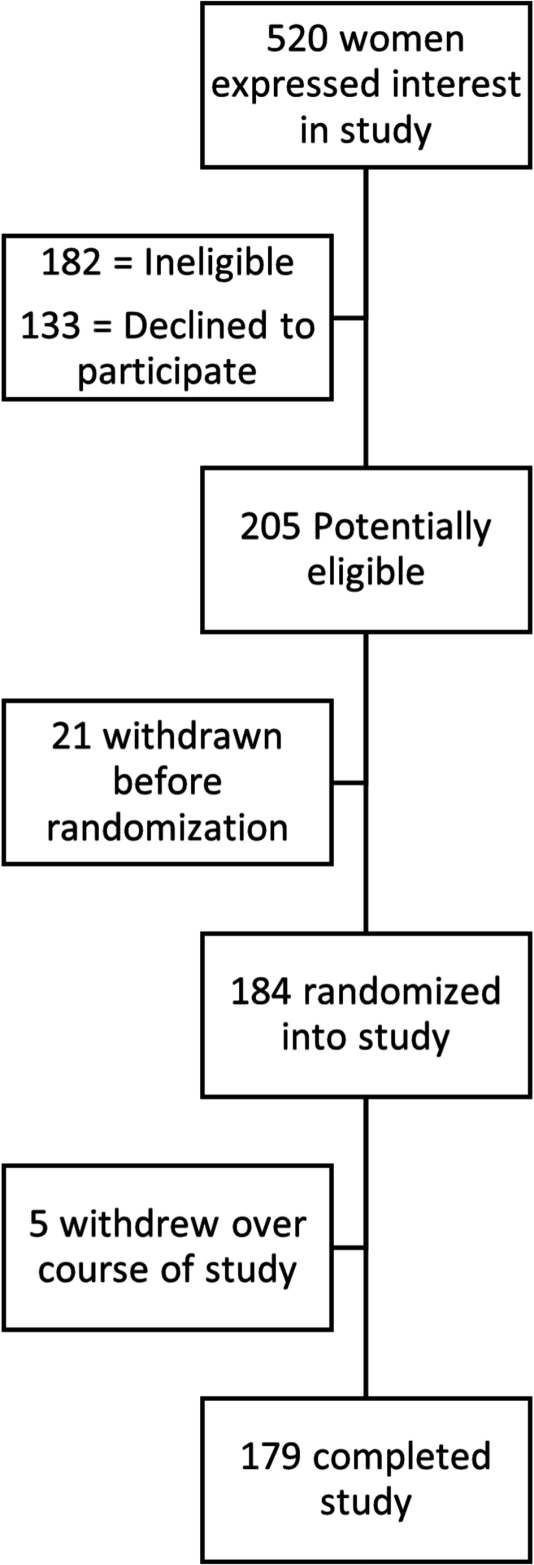
Flow of study enrollment and attrition.

Participants primarily learned about the study through social media platforms such as Facebook and Instagram (6). The study enrolled 205 participants. Of these, 21 withdrew before randomization (V1) for non-operational reasons, either volunary withdrawals or no longer meeting the trial criteria following the screening visit. Thus, 184 participants were randomized into the trial, of which, 5 (2.7%) withdrew after randomization, leaving 179 (97.3%) who completed the study. Figure 3 shows this flow of participation over the course of the study.

Among the participants who remained in the study, four missed the V2 visit (~36 weeks ‘gestation) as they gave birth before this visit, thus they progressed directly to V3, the first postpartum visit. During the visits that were conducted, only one questionnaire was not completed (1 Early Feeding Questionnaire at V3). Biological sample collection rates were high for both saliva (98%) and stool samples (93%). There were 11 missing stress measurements using the digital app (99% complete), the majority of which were due to technical difficulties with the app when a new iOS version release came out mid-study. Taken together, this resulted in an overall quite complete data set for analysis.

Participants were generally compliant with investigational product intake, with 162 of the participants (91%) consuming the product on at least 80% of study days. There were two major protocol deviations reported which were both related to poor investigational product intake and they were excluded from the per protocol analysis.

### Qualitative assessment of participant experience

The overall participant characteristics for the trial has been previously published (6). For the qualitative study, a subset were invited for follow-up interviews.

#### Overall study experience

Overall, the study participants reported having had a positive experience in participating in the trial. They also expressed a preference for the decentralized trial approach, as it was perceived to be both more convenient and safer during the COVID-19 pandemic than a traditional, in-person study. Some example verbatims from the participants have been provided below:

Convenience:

*“All I have to do is just put aside some of my time. I think the fact that everything has been given to me, even the [sample-collection materials and all] we have to do is just wait for someone to collect [it]. It was super easy so I had no trouble with it.” (30–34 years old, multiparous).*

*“I think I love it. It saves time. It saves everything and we can just … hold the time, we do not really need to prepare earlier, or have to go out. You do not have to travel. So, it’s really convenient.” (30–34 years old, primiparous).*

Safety:

*“I would [sic] actually felt safer during this COVID time. You see I do not have to come down and there’s no physical contact. … I’m alright with chatting with you online.” (30–34 years old, multiparous).*

#### Experience with recruitment process

The recruitment method targeting social media made the participants feel special as they felt that not everyone would see the advertisement on Instagram or Facebook. Participants found advertisements targeting pregnant women resonated with their current state and wanted to support science on this topic. Some also mentioned interest in the topic of probiotics in general, and hoped these might benefit several aspects of their health, in addition to the topic of mood.

Social media for recruitment:

*“I was scrolling through Instagram stories, they will pop up once in a while. I did not see it like just once or twice I saw it like 5, 6 times. Now it was like OK fine, I need to join this… guess maybe ‘cause. I see a lot of times. You know, I believe in fate.” (20–24 years old, primiparous).*

*“I was scrolling through Instagram and I … saw this ad looking for, you know, pregnant mummies to get on board on this test and thinking, ‘well, I’m pregnant’. You know this is interesting. I’ve never contributed much to science before. Anything. Yeah. So I thought, OK, let us give it a go.” (30–34 years old, multiparous).*

Appeal of probiotics:

*“Maybe because I thought that if I happen to be part of the sample group, that could have the probiotics and be good for my health as well… I guess in terms of like helping me to have better bowel movements. It was like at that time I heard that pregnant women have a lot of constipation problems, which turns out to be true.” (25–29 years old, primiparous).*

The study being conducted by A*STAR, a respected government research organization in Singapore, gave credibility to the legitimacy of the study and convinced prospective participants that it was safe to participate. They were also comfortable sharing health information with such an organization and trusted that their data would be handled safely.

*“Because if it’s a government body I sort of thought OK quite interesting why not try it” (35–40 years old, primiparous).*

“I guess I’m comfortable because the study is an A*STAR study. And when I saw the consent form is very comprehensive… I think is linked to the government. Yeah, I heard they do a lot of research, a lot of scientific studies. Even though it’s my first time doing a clinical study, but because it is A*STAR, it should be OK, I do not need to worry about.” (30–34 years old, multiparous).

In our study, we have also observed that the participants wanted additional reassurance that the probiotic was safe to be taken during pregnancy. This was a common question the study team received during the recruitment process, so we prepared additional materials with the safety data and references for the study team to use when discussing with prospective participants.

*“My concern was I did not know whether it was safe… for pregnant women to take probiotics at that moment.” (25–29 years old, primiparous).*

*“[My concerns were mainly about] the product. The study product that I’ll be taking. And how would it be… whether it would be of any harm. Whether it’s a product taken by other people before or is it highly experimental.” (25–29 years old, primiparous).*

Women wanted to discuss with their spouses before confirming that they would join the study. The most common reason given for women to decline to participate in the study (*n* = 40) as well as the second most frequent reason for withdrawal between the screening visit and visit 1 (*n* = 3) was husbands who were not supportive of participation in the trial. It seems that participating in a clinical trial during pregnancy is often decided as a family unit.

*“Oh, I discussed with my husband first. Yeah, and ask for his opinion before I registered as a participant.” (25–29 years old, primiparous).*

*“As far as I remember, I wanted to sign [up] immediately, but also [wanted to] inform my husband about it.” (30–34 years old, primiparous).*

#### Technical aspects of remote participation

Participants were comfortable using the different smartphone apps required to participate remotely in the trial. These included apps for completing surveys, for video meetings, and for collecting stress measurements using the phone camera. Even participants who encountered technical issues along the way were not very concerned, as people are used to app issues in everyday life.

“*It was all on my mobile phone, which is another thing that I like because I did not need to like grab my laptop or have to set up multiple devices. It was just on one device.” (20–24 years old, primiparous).*

*“I was like, ‘oh my God, this is so cool’. You can study my stress level just by looking at my face.” (30–34 years old, multiparous).*

*“Sometimes the apps were not working that well. But thankfully I managed to give you feedback and I was able to uninstall and reinstall and then … I could get all the results.” (35–40 years old, primiparous).*

Participants were happy with completing the study on their own personal phone, and preferred this to the idea of borrowing a device with apps pre-installed. They like the idea of using a familiar phone and wanted to avoid carrying and charging an extra device, as well as the responsibility for a borrowed device.

*“I’m just fine to use my phone, there’s no issue with my phones and everything up to date where I can download the apps. I know sometimes on different phones might have issues with different apps. … I probably would prefer using my phones over the device that you we are going to send me because in that case I will we have to use two devices and I might miss out any notifications. … [It also might take longer to reach me] because I probably will not be looking at that additional device all the time. We probably have to charge it also.” (30–34 years old, primiparous).*

*“[If I borrowed a device] I’ll be very paranoid, like if I do not charge it now, then what if the battery drains? And then while the battery spoils, you know, then I will return a device that is you know, worse off condition than when it came to me. In the same sense, but I’ll be afraid of like, what if I drop it? What if I spoiled that device? Then I have to pay them back. You know, it’s like an added [source] of stress for me, I guess.” (20–24 years old, primiparous).*

*“I would probably think it’s easier for me just to do everything my phone because then I have to worry about, you know, making sure I do not spoil the devices, have to return it and make sure my kids do not take it at night, play with it, etc. Right. So there’s one more thing to worry about, so I’d rather not have that ‘cause I mean it wasn’t difficult for me to use [my] phone.” (35–40 years old, multiparous).*

#### Challenges in stool collection

One pain point of trial participation was around needing to store stool samples in the freezer if they could not be picked up on the same day; for example, when they were collected late at night. Participants mentioned needing to “sneak around” to store their stool samples in the freezer without disgusting other members of their household. They suggested that future studies explore options for faster/overnight pickup of stool samples or provide a cooler or mini-fridge to avoid needing to use the family freezer.

*“Yeah, I had to hide it lah. Basically, I had to. I had a dedicated entire section of my freezer [for my] sample and then I had to like, quietly hide it in the freezer so my husband would not be so hypervigilant and aware of the fact that sample was there” (30–34 years old, primiparous).*

*“There was once that my father was in the kitchen, and then I had collected my stool sample already. I was like, my father is outside, I cannot put it in the freezer now. What if he sees me, you know. I was waiting for him to, like, go off first before I went and I put it in the container and then in a plastic bag, which I stuffed into the fridge.” (20–24 years old, primiparous).*

*“I do not want to put any of the fecal matter into my fridge, but at least there [could be a] cooler bag provided so I can put it in the cooler bag instead” (35–40 years old, primiparous).*

Some participants also mentioned that as constipation can be a common issue during pregnancy, a wider window to collect the stool samples could be helpful in future studies.

“*[at the end of the study] I felt relieved that I do not have to collect anymore stool samples, haha…. ‘cause it was it was quite a little stressful for me because I sometimes have a bit of constipation problems, so I was a bit worried that I may not be able to collect my samples on the date.” (25–29 years old, primiparous).*

*“I will say that it has some difficulties because you have to collect a few samples on different occasions, for example for the stool, I think it can be challenging. If it’s like I want to plan it for the next day, but I may not have the feeling of passing motion or, constipation issue” (35–40 years old, primiparous).*

### Environmental impact of the clinical operations

**Figure 4:**
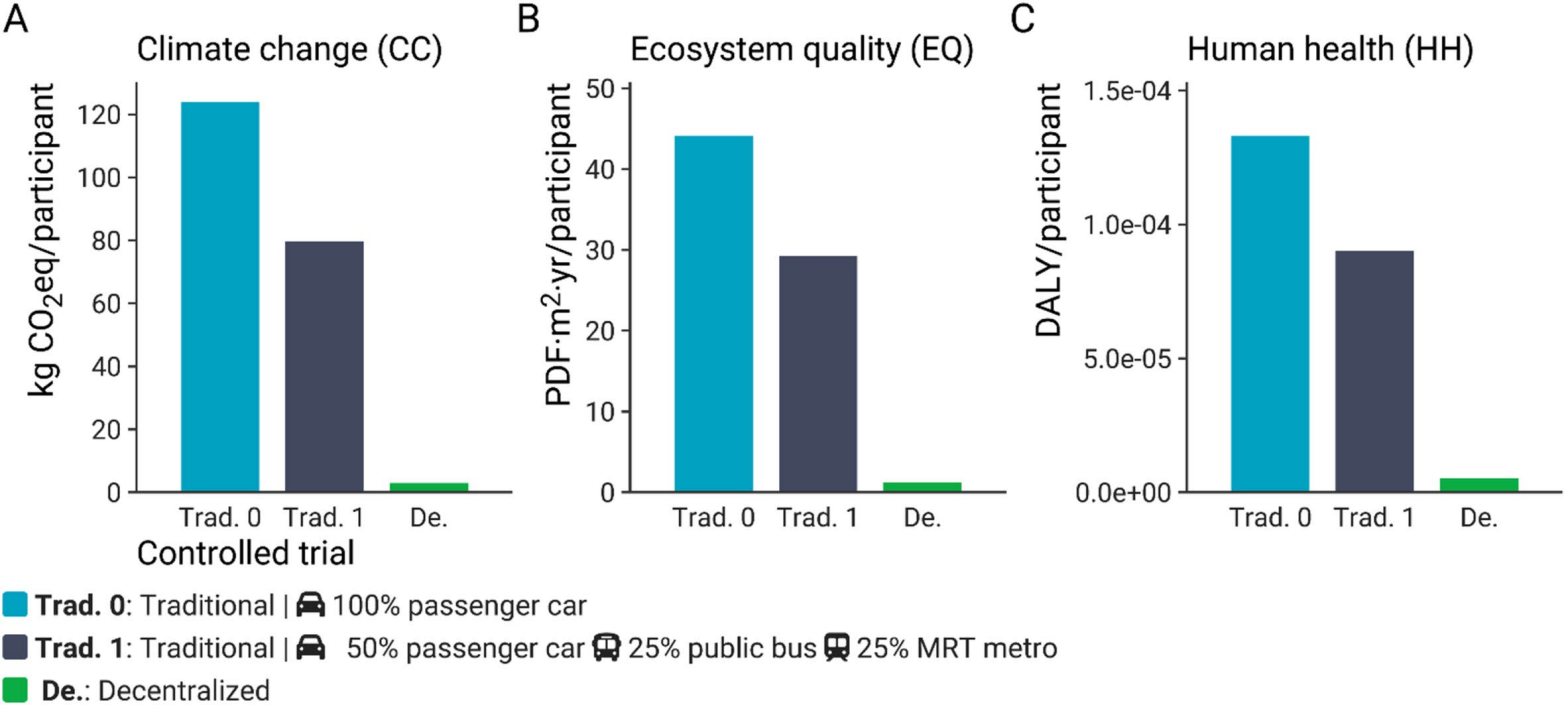
Climate change (CC) impacts (kg CO_2_eq/participant) **(A)**, ecosystem quality (EQ) impacts (PDF·m^2^·yr./participant) **(B)**, and human health (HH) impacts (DALY/participant) **(C)** of a clinical trial carried out according to a traditional (Trad. 0 – Passenger car only), traditional including public transport (Trad. 1), or decentralized model. The impact scores were calculated with IMPACT World + method (2.0 V2.00) (11).

Figure 4 shows the environmental impact of traditional (both car-only and with public transit scenarios) or entirely decentralized clinical operational models for outcomes related to short-term climate change (kg CO_2_eq/participant), ecosystem quality (PDF·m^2^·yr./participant), and human health (DALY/participant). The carbon footprint, measured in terms of the climate change (CC) short-term indicator, of a traditional clinical trial is 123.9 kg CO_2_eq/participant. In contrast, the carbon footprint of a decentralized clinical trial is approximately 3.0 kg CO_2_eq/participant. Thus, the carbon footprint of a traditional clinical trial is around 41 times greater than that of a decentralized trial (Figure 4A). Similar sharp differences between the potential impacts on ecosystem quality and human health were observed. The ecosystem quality impact score associated with a traditional clinical trial is around 44.1 PDF·m^2^·yr./participant, which is approximately 37-fold the score calculated for a decentralized trial (1.2 PDF·m^2^·yr./participant) (Figure 4B). Regarding the impacts on human health, a potential impact of 1.33 × 10^−4^ DALY/participant was computed for a traditional clinical trial, which is around 26-fold times the value estimated for a decentralized clinical trial (Figure 4C). To contextualize these differences, accepted LCA criteria suggest that a difference of more than 10% is considered significant for climate change impact scores. For particulate matter formation and acidification, a difference greater than 30% is deemed significant, and for toxicity categories, a difference of one order of magnitude (a factor of 10) is necessary (16). These categories are the main contributors to the human health and ecosystem quality endpoints in this study (Supplementary Figure S3). While we acknowledge that a more detailed analysis of uncertainty propagation, such as through a Monte Carlo simulation, could assess the statistical significance of the observed differences for human health impacts and ecosystem quality, SimaPro v9.3.0.3 software does not support the inclusion of uncertainty in characterization factors.

Furthermore, the analysis of the impact categories at endpoint level confirmed that the clinical trials conducted in a decentralized model were better positioned regardless of the environmental indicator considered. The normalized values for the impact categories contributing to ecosystem quality for a decentralized clinical trial are under 7% compared to a traditional clinical model. Similarly, the normalized scores of the impact categories leading to human health impacts are at most 9.5% compared to the corresponding scores for a traditional clinical trial (Supplementary Figure S1).

**Figure 5:**
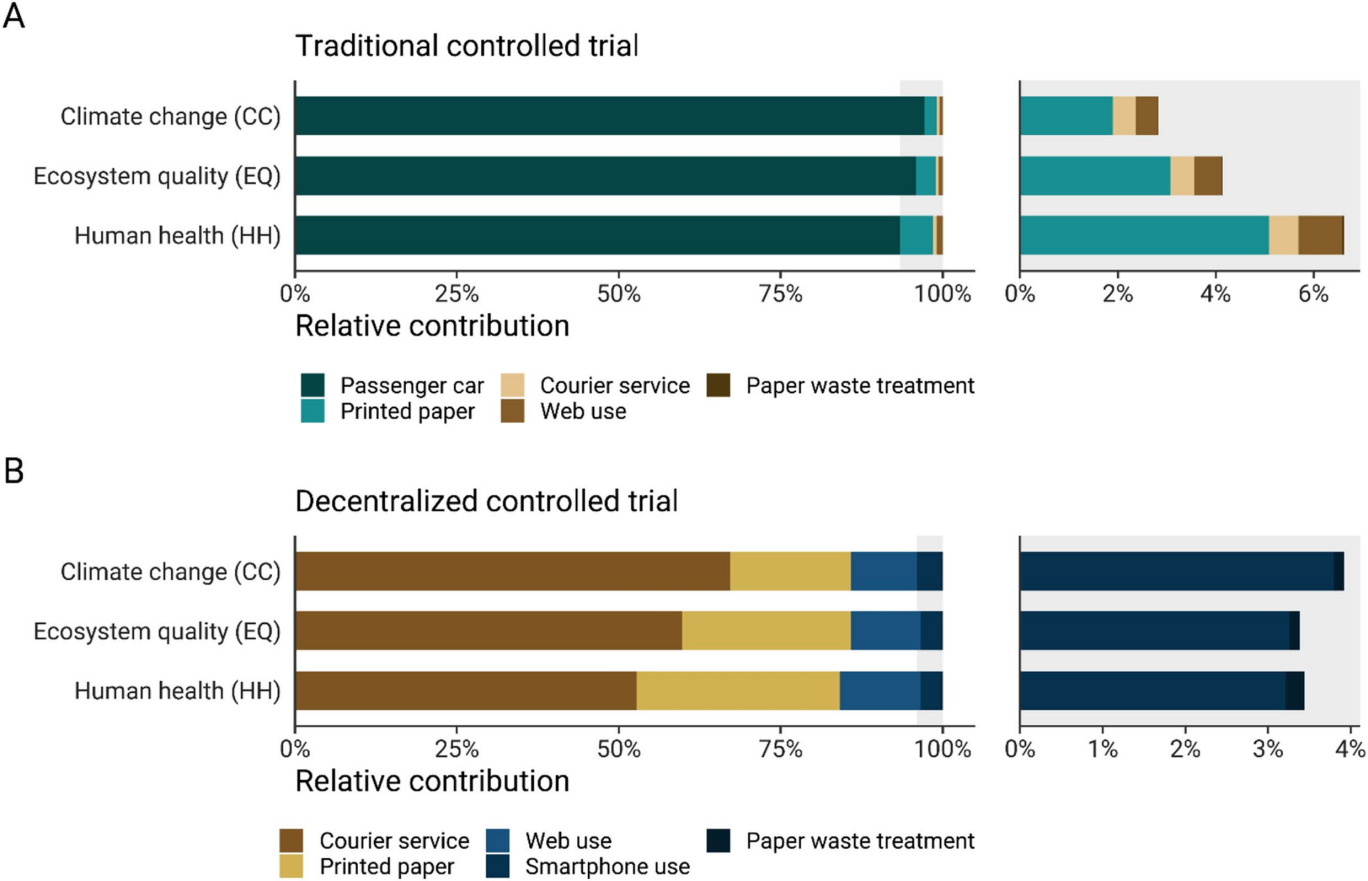
Relative contribution (%) of each process to climate change (CC) impacts (kg CO2eq/participant), ecosystem quality (EQ) impacts (PDF·m2·yr./participant), and human health (HH) impacts (DALY/participant) of a clinical trial carried out according to a traditional **(A)** or decentralized model **(B)**. The right side of the graph shows a zoomed-in view of the factors that contribute less than 7%. The impact scores were calculated with IMPACT World + method (2.0 V2.00) (11).

Figure 5 presents a breakdown of the main contributors to the environmental impacts of a clinical trial performed in a traditional and decentralized model. The breakdown for the traditional model shows that the passenger car is the major contributor to the impact indicators considered in this study: 97.2% of climate change impacts, 95.9% of ecosystem quality impacts, and 93.4% of the potential impacts on human health (Figure 5A). The human health impacts associated with passenger car use are primarily driven by both the production and operation phases of the vehicle, which contribute mainly to particulate matter formation and human toxicity (Supplementary Figure S3). Specifically, the impacts on particulate matter formation are predominantly influenced by the emissions of fine particulate matter (PM_2.5_), sulfur dioxide (SO_2_), nitrogen oxides (NOx), and ammonia (NH_3_). In a distant second place is the environmental impacts of the printed paper used in a traditional trial, which contributes to around 5.1% of the potential impacts on human health, 3.1% of impacts on ecosystem quality, and less than 2% to climate change. In the event the participant is unable to provide fresh stool samples on the day of the visit, courier services would still be required. However, the contribution analysis shows that the courier service for collecting biological samples is not an important contributor to the impact of a traditional clinical trial. It contributes 0.5–0.6% to the impact across the indicators considered (Figure 5A). Furthermore, the contribution of web access and paper waste treatment to the impact categories is negligible (less than 0.9%). In a decentralized clinical trial, it was found that the main contributor to the impact indicators is the courier service, which contributes 67.1% to climate change impacts, 59.8% to ecosystem quality impacts, and 52.7% to human health. The second principal contributor to the potential environmental impacts is the use of paper for the instruction booklets, contributing around 18.7% of climate change impacts, 26.1% to ecosystem quality, and 31.4% to potential impacts on human health. In this model, access to the internet also has a greater relative contribution to the impact categories compared to the traditional scenario. Access to the internet contributes to around 10.2–12.4% to climate change, ecosystem quality, and human health. Furthermore, the use of a smartphone to access the app in which a participant completes follow-up questionnaires contributes to around 3.4% of the total impact of a decentralized clinical trial across the considered indicators. Finally, paper waste management had a negligible contribution to the total impact for all the impacts categories (around 0.1 to 0.2%).

### Results of scenario analysis

As cars were the main contributor to the environmental impact of a traditional clinical trial, we also ran a scenario in which some of the participants took public transport. This alternative scenario (Trad. 1) causes less potential environmental impacts compared to the reference scenario (Trad. 0). Including public transport in conducting clinical trials in a traditional model reduces the potential environmental impacts by 32 to 36% (Figure 4). For instance, the carbon footprint of a clinical trial under a traditional model would decrease from 123.9 to 79.7 kg CO_2_eq/participant when public transport is included. However, the latter carbon footprint is still around 27-fold greater than that computed for a clinical trial under a decentralized model (Figure 4).

The contribution analysis of a traditional clinical trial conducted with public transport shows that the transportation modes (passenger car, public bus and MRT metro) are still the main contributors to the potential damages: 96% of climate change, 94% of ecosystem quality, and 90% of human health, with the passenger car remaining the principal contributor to the potential impacts (69–76%), followed by the public bus (10–12%), and the metro (8–11%). The sensitivity analysis comparing the results obtained with the IMPACT World+ method (Figure 4) to those computed with ReCiPe (Supplementary Figure S2) found that both methods supported the conclusion that a decentralized clinical trial has a better environmental profile than a traditional one. The sensitivity check found a negligible difference in estimated carbon footprint between the two methods for conducting a clinical trial. This is explained by the fact that these methods derived their characterization factors from the global warming potentials (GWP-100) for a 100-year period reported by the IPCC. The impacts on ecosystem quality cannot be directly compared between the two methodologies as they use different metrics (PDF·m^2^·yr. and species·yr., respectively). The ecosystem quality impact computed with ReCiPe (H) for a traditional clinical trial is around 25 times the value computed for a decentralized clinical trial, while this ratio is approximately 3 times according to the calculated scores with IMPACT World+. Finally, the human health impact calculated with ReCiPe for a decentralized clinical trial was in perfect agreement with the scores computed with IMPACT World+; nonetheless, the computed scores for a traditional clinical trial with ReCiPe are 8% higher compared to that obtained with IMPACT World+. Overall, the two employed impact methods supported the conclusion that a decentralized clinical trial has a better environmental profile compared to a traditional one.

**Figure 6:**
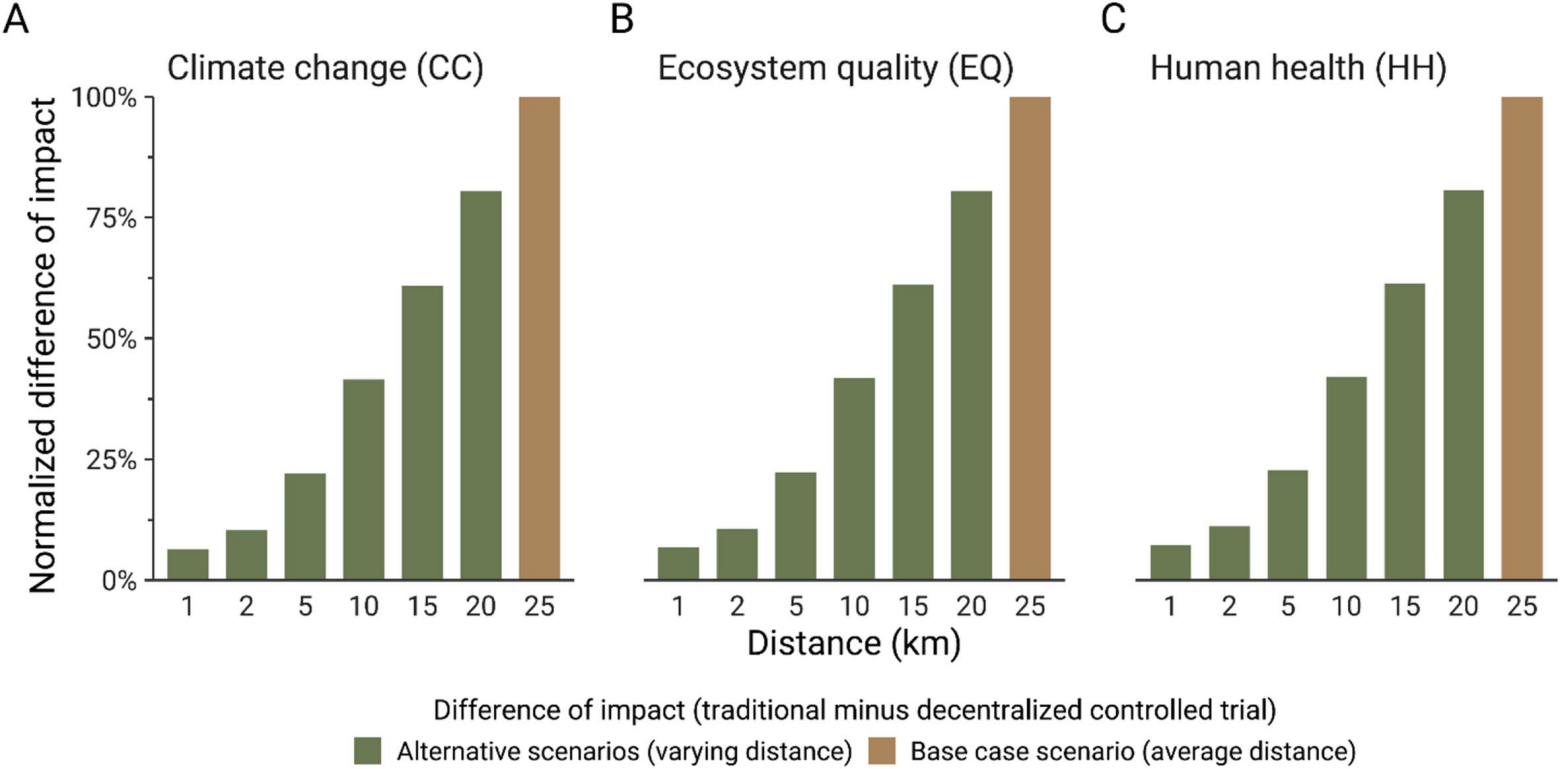
Normalized difference (%) of carbon footprint **(A)**, ecosystem quality (EQ) impacts **(B)**, and human health (HH) impacts **(C)**. The difference between the impact score of the traditional and decentralized model was normalized to the highest computed difference for each indicator. The impact scores were calculated with IMPACT World + method (2.0 V2.00) (11).

As described in Toh et al. (6), participants were recruited across 4 districts in Singapore. Therefore, to facilitate comparing traditional and decentralized models for conducting clinical trials, an average distance of 25 km was considered to model the displacement from the participant’s home to the study center, and between the participant’s home and the courier service. Given the variability of the distance of transport and the large contribution of this process to the environmental impacts of a clinical trial (passenger car for a traditional trial and courier service for a decentralized trial, Figure 6), a second sensitivity analysis aimed to test the influence of the transportation distance on the environmental impacts of clinical trials. Figure 6 shows that for all the impact indicators considered in this study, there is no breakeven point with lower transportation distance because of the high contribution of the passenger car to the potential environmental impacts of a traditional clinical trial, even though transport is also included in a remote clinical trial to account for courier services. A reduction of 5 km of transport leads to a decrease of around 20% of the carbon footprint for a traditional clinical trial and around 16% for a decentralized clinical trial. Therefore, if a study requires a traditional clinical trial approach, efforts toward promoting group transportation for each clinical visit would help reduce the impacts, such as promoting public transportation or providing a shuttle to collect several participants.

## Discussion

From an operational perspective, this decentralized clinical trial had very high participant retention rates (97% post-randomization), which is impressive considering the median retention rate (of participants providing primary outcome data) from traditional trials in general was estimated to be 89% (17). Trials covering the busy and demanding peripartum period could pose additional challenges for retention. In addition, we observed high levels of clinical data set completion, even for stool samples (93%), which tend to be difficult to collect [e.g., (18)]. The high retention rate can likely be, at least in part, attributed to the decentralized model of the trial, which allowed participants more flexibility in terms of schedule and reduced the time burden of participation as no travel time was required. There was also generally a primary point of contact within the study team that conducted all study virtual visits with each participant; this allowed a certain familiarity and rapport to develop which helped maintain participant engagement throughout the study duration.

Social media channels such as Instagram were the most successful in reaching and attracting pregnant women to join the study. This is consistent with previous studies finding that social media can be an effective recruitment strategy, especially for studies targeting adults and for online interventions (19, 20), which like ours, do not require travel to a study site. One advantage of electronic advertisement placement is that they can be targeted to a specific population or area of interest; in our case, pregnant women and pregnancy. Previous studies have also found targeted ads to be a cost-effective way to meet recruitment targets (21). A surprising finding was that the participants appreciated the “serendipity” of seeing advertisements related to pregnancy and that this attracted their attention. It also seems that the subject matter of the study, including both probiotics as an intervention and mood as an outcome, were attractive, which may have helped study recruitment. This method of recruitment also resulted in self-selection of participants who would have likely felt comfortable and are more adept with using digital tools for clinical studies. It is possible that the subject matter of mood issues may have been especially resonant during the COVID-19 pandemic. Recruitment rates were further enhanced by active monitoring and adjustment by the study team, who tracked the click-through rates of different digital channels and even specific ad designs that were posted. This made it possible to adapt the placement of study materials moving forward, increasing investments in those channels, and using the study advertisement designs that were most effective in attracting the attention of the target population. Common questions asked during the informed consent process were also noted, and more detailed materials about key topics of concern (e.g., questions around the safety of the experimental product during pregnancy) were prepared to facilitate discussions with future study candidates.

Participants generally found the decentralized clinical operation model experience to be convenient and welcomed the additional safety of being able to participate from home during the pandemic. Participants in this study were tech-savvy women who were comfortable with digital apps, especially when using their personal mobile phone to enter study data. The simplicity and user-friendliness of the interface to enter data is very important, and if participants are asked to interact with multiple systems (ePRO, multiple devices, electronic health records, etc.), this increased complexity could be dissuasive to trial completion.

The decentralized clinical operation model, as evaluated in the current simulation approach, was found to be more environmentally friendly and less detrimental to human health than a traditional face-to-face clinical trial. Given that the courier service was the main contributor to the impact indicators for the decentralized study, using an in-house trained and licensed courier for biological sample transport, or a courier service with a base located close to the study center could have further reduced the environmental impact of the study. The environmental impact of the courier service will also vary depending on the number of parcels to be delivered and the efficiency of their route planning, which may be out of the control on the clinical team and can be difficult to estimate for such analyses. However, clustering deliveries when possible (e.g., picking up all of the sampling kits for the week in one trip) can help reduce the number of displacements required. Importantly, reducing participant usage of cars (personal or taxis) for site visits was found to be the main driver of the decreased environmental impact compared to traditional clinical trials. Paperwork was not a significant contributor to the impact of traditional clinical trials. We acknowledge that there were some conservative assumptions applied to the LCA models. For instance, we used an average transportation distance in our calculations. However, our sensitivity analyses testing a range of distances showed no break-even point, suggesting that the decentralized model will always perform better if the participants live far enough away that they need to travel by car. Further, we recognized that under normal, non-pandemic situations, many Singaporeans would have taken public transport rather than driving. Thus, we conducted an alternative scenario analysis to demonstrate that even when half of the participants took the bus or metro to a study site for a traditional clinical trial, the decentralized study still performed better. The current model assumes that drop-out rates would be similar between in-person studies and remote studies, but based on past experience and participant interview responses, we suspect that the remote study would have had better retention rates. Thus, an in-person study might have had additional environmental impacts from participants who would have been over-recruited and dropped out with incomplete data.

This is the first time that a life cycle assessment model was applied to evaluate the environmental impact of clinical operations. Although this model was developed after our trial execution, we do believe that this model could be used in the future during the design/set-up phase of a trial to optimize the environmental impact of the clinical operations. While conducting the current analyses, we identified areas in which our own study could have been improved from an environmental perspective, such as by combining pick-ups and drop-offs of study materials to reduce the number of courier trips. Of course, the environmental aspect should not be the only driver, elements such as participant safety, convenience, data quality must also be considered in the overall equation.

In addition to being more environmentally friendly, it is possible that decentralized clinical operations may reduce study costs as well. If the current participant retention figures (97%) are representative, it is possible that future decentralized studies could recruit fewer participants than traditional in-person studies, reducing the recruitment duration and burden on the study team. With rising fuel and transportation costs (22), participants may be even less willing to travel to study sites in the future or could require more travel compensation, which would increase study costs further or make recruitment even more challenging. Limiting the amount of physical travel required and/or providing participants with convenient options and travel compensation favoring public transit would also be consistent with current trends for a “greener” lifestyle (23).

In both traditional and decentralized clinical trials, there is a trend toward more digitalized data collection, such as by having participants complete questionnaires on a phone or computer rather than on paper (24). In addition to reducing paper waste, there are other advantages of collecting primary data digitally. One important benefit is reducing the need for data entry from the paper questionnaire into a study data file, which is both time consuming for research staff and introduces additional opportunities for human errors such as typos that can impact data quality. Digitally collected data can also be monitored in real-time, making it possible to catch errors or seek clarification quickly (24), rather than trying to decipher or ask participants to recall how they responded when problems are identified when data is entered at the end of the study. Although it can be tempting to digitize as many measures as possible, it is important to ensure that the measures have been validated in digital format. Just as results may differ whether responses are collected via an interview or a written questionnaire (25, 26), the transition from a validated paper questionnaire to digital formats is not always straightforward (27), and particular care may be needed when adapting for presentation on mobile devices to avoid potential biases.

No study design is perfect and decentralized clinical studies also face challenges that should be carefully considered when the operational approach is defined. At a very fundamental level, not all clinical trial measures can be collected remotely: if a study requires specialized medical examinations (e.g., an MRI), this simply must be done at a designated facility. However, in many cases, at least a hybrid approach could be considered, with some measures collected on-site and others (e.g., questionnaires) from the comfort of home. Other studies may opt to specifically select measures that are more convenient to collect remotely, but the convenience/decentralization aspect needs to be considered in the context of the strength of evidence and validity of each measure. Although questionnaires can easily be collected remotely, it can be difficult to assess whether participants are answering seriously, or even if the person responding is who they say they are (e.g., one participant filling in questionnaires on behalf of other household members). In the current study, study “visits” were conducted by video chat, where we could see the participant and questionnaires were completed “live” during this session, which allowed the participant to ask questions if anything was unclear. For studies collecting questionnaire responses through a web portal, some platforms provide data on the amount of time spent completing a survey, which could be considered for excluding responses that were submitted too quickly for participants to have read and responded thoughtfully.

Before deciding to conduct a decentralized clinical trial, it is important to assess the “readiness” of the site, infrastructure, and population to conduct such a trial. The current study was conducted in Singapore, a country with strong digital infrastructure, broad penetration of internet-enabled devices, and a tech savvy population, especially as our study focused on women of childbearing age. Studies in countries with less developed infrastructure or in older adult populations may need to consider whether this approach is a good fit, and whether recruitment based on such criteria would still be representative of the target population. Decentralized trials require a high level of digitalization and the data collected needs to be integrated from several sources. This complex data architecture may influence the end-to-end data flow and integration. Careful upfront planning of data flow and database structure can help to avoid unwieldy workflows, data errors, and study delays. It is also critical the methods of collecting and storing participant data are appropriate for protecting sensitive health information. Further, it may be necessary to explain these data protection measures to participants to reassure them that their health data will be kept secure. Participants may vary in their level of comfort with sharing personal data. In the current study, we found that our participants in Singapore tended to be trusting of government organizations to protect their data, which facilitated recruitment; however, in other populations prospective participants may be more wary of sharing electronic data.

Decentralized clinical trials also require a change of mindset and the development of new skills by study staff. As for any innovation, some people may feel uncomfortable or anxious when disrupting the legacy clinical operations approach. Technology is not always intuitive, and it will be important to assess the readiness of both the study staff and target population to adapt digital technologies and tools. Providing appropriate staff training and clear instructions for participants can help smooth this transition. We recommend accounting for additional time during the startup phase of the project to pilot-test any digital tools to ensure user acceptance and that the instructions are clear. It is also important to have readily available technical support staff who can help participants when problems arise that require troubleshooting. Delays in resolving issues can result in lost data (e.g., missing the appropriate window to collect outcome data) and create challenges for retaining participant engagement, thus it is imperative that problems can be addressed quickly and efficiently. In the current study, participants were asked to use their own devices for these assessments, rather than providing a device for them to use, and indicated preferring using their own device due to the familiarity and convenience. Providing a standardized device for all participants may have other advantages, such as reducing the risk of inconsistencies between software versions. For studies in which participants use their own devices, it is important to ensure the software is compatible for all platforms and that major app updates will not happen during the study to interruptions to data collection.

The local regulatory environment may affect whether and which aspects of a study can be decentralized. For example, in the United States, the digitization of many clinical study steps is generally accepted and supported by the FDA (28). In other countries, the adaptation is more reserved and regulations can vary widely. Before planning a decentralized clinical study, a careful conversation with ethics committees in each country is highly recommended. Beyond legal requirements, it may be more difficult to convince participants that the study is legitimate without the “white coat” effect of physically visiting a university or hospital. In the current study, prospective participants were reassured of the credibility and safety of joining the study, based on the reputation and government affiliation of the study coordinators. Using official channels for participant communication, such as university websites for providing study information and official e-mail addresses for participant correspondence can help build trust.

In a post-Covid19 era marked by an RCT landscape that is increasingly evolving toward more decentralized and hybrid trial designs, we hope this study and its learnings will inspire further research to use this approach to optimize the environmental footprint of clinical studies. Environmental sustainability is yet another relevant dimension to be taken into account for the selection of the optimal model to conduct a trial. It must be accompanied with a thorough assessment of the readiness for decentralized trial operations across the value chain, including participant and site skills, and the regulatory and digital maturity of the country where the trial will be carried out.

## Conclusion

The decentralized clinical trial model achieved impressive participant retention rates (97%) and high completion rates for clinical data, even for challenging sample collection. The flexibility and reduced time burden of participation, along with a primary point of contact within the study team, contributed to the high retention rate and participant engagement. Participants found the decentralized model convenient and safe, especially during the pandemic.

Moreover, the decentralized model was found to be more environmentally friendly and less detrimental to human health compared to traditional face-to-face clinical trials, primarily by reducing participant usage of cars for site visits. While this study focused on the environmental impact, it is important to consider other factors such as participant safety, convenience, and data quality when evaluating the suitability of a decentralized clinical trial approach. Careful planning of data flow, database structure, and data protection measures is essential. Additionally, a change of mindset and the development of new skills by study staff are necessary to adapt to the digital technologies and tools used in decentralized trials.

This study contributes to the growing knowledge on optimizing the environmental footprint of clinical trials. Environmental sustainability, along with other factors, should be evaluated when selecting trial models.

In a post-COVID-19 era, decentralized and hybrid clinical trials offer efficiency, effectiveness, and environmental benefits. Further research and adoption of this approach are encouraged.

## Data Availability

The raw data supporting the conclusions of this article will be made available by the authors, without undue reservation.

## Footnotes

^1^clinicaltrials.gov registration # NCT04685252.

## References

[1] Jean-Louis G Seixas AA. The value of decentralized clinical trials: inclusion, accessibility, and innovation. Science. (2024) 385:eadq4994. doi: 10.1126/science.adq4994, PMID: 39172847

[2] Petrini C Mannelli C Riva L Gainotti S Gussoni G. Decentralized clinical trials (DCTs): a few ethical considerations. Front Public Health. (2022) 10. doi: 10.3389/fpubh.2022.1081150, PMID: 36590004 PMC9797802

[3] Margas W Wojciechowski P Toumi M. Impact of the COVID-19 pandemic on the conduct of clinical trials: a quantitative analysis. J Mark Access Health Policy. (2022) 10:2106627. doi: 10.1080/20016689.2022.2106627, PMID: 35968522 PMC9367669

[4] Medidata and Vanson Bourne. (2022). European industry research report: the future of clinical trials. Available online at: https://www.medidata.com/en/european-industry-research-the-future-of-clinical-trials/

[5] Suman A van Es J Gardarsdottir H Grobbee DE Hawkins K Heath MA. A cross-sectional survey on the early impact of COVID-19 on the uptake of decentralised trial methods in the conduct of clinical trials. Trials. (2022) 23:856. doi: 10.1186/s13063-022-06706-x, PMID: 36203202 PMC9535935

[6] Toh MPS Yang CY Lim PC Loh HLJ Bergonzelli GE Lavalle L. A probiotic intervention with *Bifidobacterium longum* NCC3001 on perinatal mood (PROMOTE; PRobiotic on MOThErs’ mood and stress): protocol for a decentralized randomized controlled trial. JMIR Res. Protocols. (2023) 12:e41751. doi: 10.2196/41751, PMID: 37018024 PMC10131660

[7] Zigmond AS Snaith RP. The hospital anxiety and depression scale. Acta Psychiatr Scand. (1983) 67:361–70. doi: 10.1111/J.1600-0447.1983.TB09716.X, PMID: 6880820

[8] Signorell A Saric J Appenzeller-Herzog C Ewald H Burri C Goetz M. Methodological approaches for conducting follow-up research with clinical trial participants: a scoping review and expert interviews. Trials. (2021) 22:961. doi: 10.1186/s13063-021-05866-6, PMID: 34961543 PMC8711196

[9] ISO. International Organization for Standardization (ISO) standards 14040: environmental management, life cycle assessment. 2nd ed. Geneva: ISO (2006).

[10] ISO. International Organization for Standardization (ISO) standards 14044: environmental management, life cycle assessment. Geneva: ISO (2006).

[11] Bulle C Margni M Patouillard L Boulay A-M Bourgault G De Bruille V. IMPACT world+: a globally regionalized life cycle impact assessment method. Int J Life Cycle Assess. (2019) 24:1653–74. doi: 10.1007/s11367-019-01583-0 PMC7592718 33122880

[12] Hellweg S Benetto E Huijbregts MA Verones F Wood R. Life-cycle assessment to guide solutions for the triple planetary crisis. Nature Rev Earth Environ. (2023) 4:471–86. doi: 10.1038/s43017-023-00449-2

[13] Fantke P. Bijster M. Guignard C. Hauschild M. Huijbregts M. Jolliet O.. (2017). USEtox 2.0: Documentation (Version 1).

[14] Huijbregts MA Steinmann ZJ Elshout PM Stam G Verones F Vieira M. ReCiPe2016: a harmonised life cycle impact assessment method at midpoint and endpoint level. Int J Life Cycle Assess. (2017) 22:138–47. doi: 10.1007/s11367-016-1246-y

[15] Department of Statistics Singapore. (2023). Usual mode of transport among resident students/working persons travelling to school/work dashboard. Available online at: https://www.singstat.gov.sg/find-data/search-by-theme/population/mode-of-transport/visualising-data/mode-of-transport-dashboard

[16] Humbert S Rossi V Margni M Jolliet O Loerincik Y. Life cycle assessment of two baby food packaging alternatives: glass jars vs. plastic pots. Int J Life Cycle Assess. (2009) 14:95–106. doi: 10.1007/s11367-008-0052-6

[17] Walters SJ dos Anjos B Henriques-Cadby I Bortolami O Flight L Hind D. Recruitment and retention of participants in randomised controlled trials: a review of trials funded and published by the United Kingdom Health Technology Assessment Programme. BMJ Open. (2017) 7:e015276. doi: 10.1136/bmjopen-2016-015276, PMID: 28320800 PMC5372123

[18] Torok M White A Butterfield M Weiss J Scallan Walter E Hewitson I. Barriers to stool specimen collection during foodborne and enteric illness outbreak investigations in Arizona and Colorado. J Food Prot. (2023) 86:100012. doi: 10.1016/j.jfp.2022.11.004, PMID: 36916595

[19] Darmawan I Bakker C Brockman TA Patten CA Eder M. The role of social Media in Enhancing Clinical Trial Recruitment: scoping review. J Med Internet Res. (2020) 22:e22810. doi: 10.2196/22810, PMID: 33104015 PMC7652693

[20] Topolovec-Vranic J Natarajan K. The use of social Media in Recruitment for medical research studies: a scoping review. J Med Internet Res. (2016) 18:e286. doi: 10.2196/jmir.5698, PMID: 27821383 PMC5118584

[21] Alley S Jennings C Plotnikoff RC Vandelanotte C. An evaluation of web-and print-based methods to attract people to a physical activity intervention. JMIR Res Protoc. (2016) 5:e94. doi: 10.2196/resprot.4826, PMID: 27235075 PMC4902856

[22] Tan C. (2022). Shell, Caltex raise pump prices as oil creeps up. Straits Times. Available online at: https://www.straitstimes.com/singapore/transport/shell-raises-pump-prices-as-oil-creeps-up

[23] Analysis.org. (2023). The shift away from private vehicle ownership: why younger generations Lead the trend. Available online at: https://analysis.org/the-shift-away-from-private-vehicle-ownership-why-younger-generations-lead-the-trend/

[24] Inan OT Tenaerts P Prindiville SA Reynolds HR Dizon DS Cooper-Arnold K. Digitizing clinical trials. NPJ Digital Med. (2020) 3:101. doi: 10.1038/s41746-020-0302-y, PMID: 32821856 PMC7395804

[25] Bowling A. Mode of questionnaire administration can have serious effects on data quality. J Public Health. (2005) 27:281–91. doi: 10.1093/pubmed/fdi031, PMID: 15870099

[26] Cook C. (2010). Mode of administration bias. 18, pp. 61–63. New York: Taylor & Francis. 10.1179/106698110X12640740712617 PMC3101072 21655386

[27] Alfonsson S Maathz P Hursti T. Interformat reliability of digital psychiatric self-report questionnaires: a systematic review. J Med Internet Res. (2014) 16:e268. doi: 10.2196/jmir.3395, PMID: 25472463 PMC4275488

[28] Food and Drug Administration. (2023). Decentralized clinical trials for drugs, biological products, and devices (FDA-2022-D-2870). Rockville, MD. Available online at: https://www.fda.gov/regulatory-information/search-fda-guidance-documents/decentralized-clinical-trials-drugs-biological-products-and-devices

[29] Hijmans RJ. Boundary, Singapore, 2015. [Shapefile]. University of California, Berkeley. Museum of Vertebrate Zoology. Available at: https://purl.stanford.edu/pg798kr1205, PMID: 15870099

