# Supplementary figures and images for: Decentralized clinical trials are better for the participants and for the planet: the case study of a double-blind randomized controlled trial in Singapore (PROMOTE study)
Source: Front Public Health. 2025 Jan 13;12:1508166. doi: 10.3389/fpubh.2024.1508166 (PMC11769950; doi:10.3389/fpubh.2024.1508166)

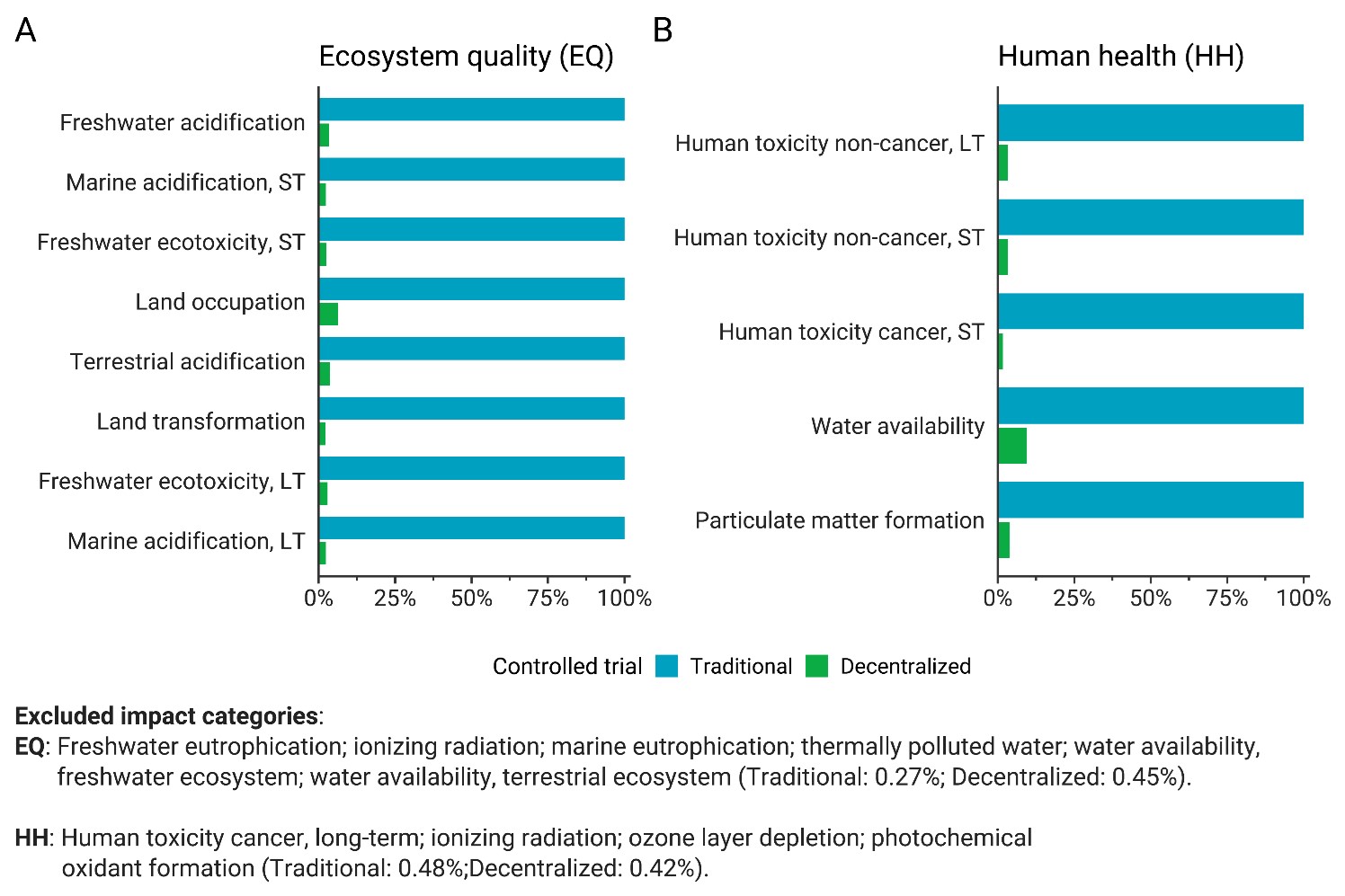

Supplement: Supplementary file 1 [file Image_1.jpg]

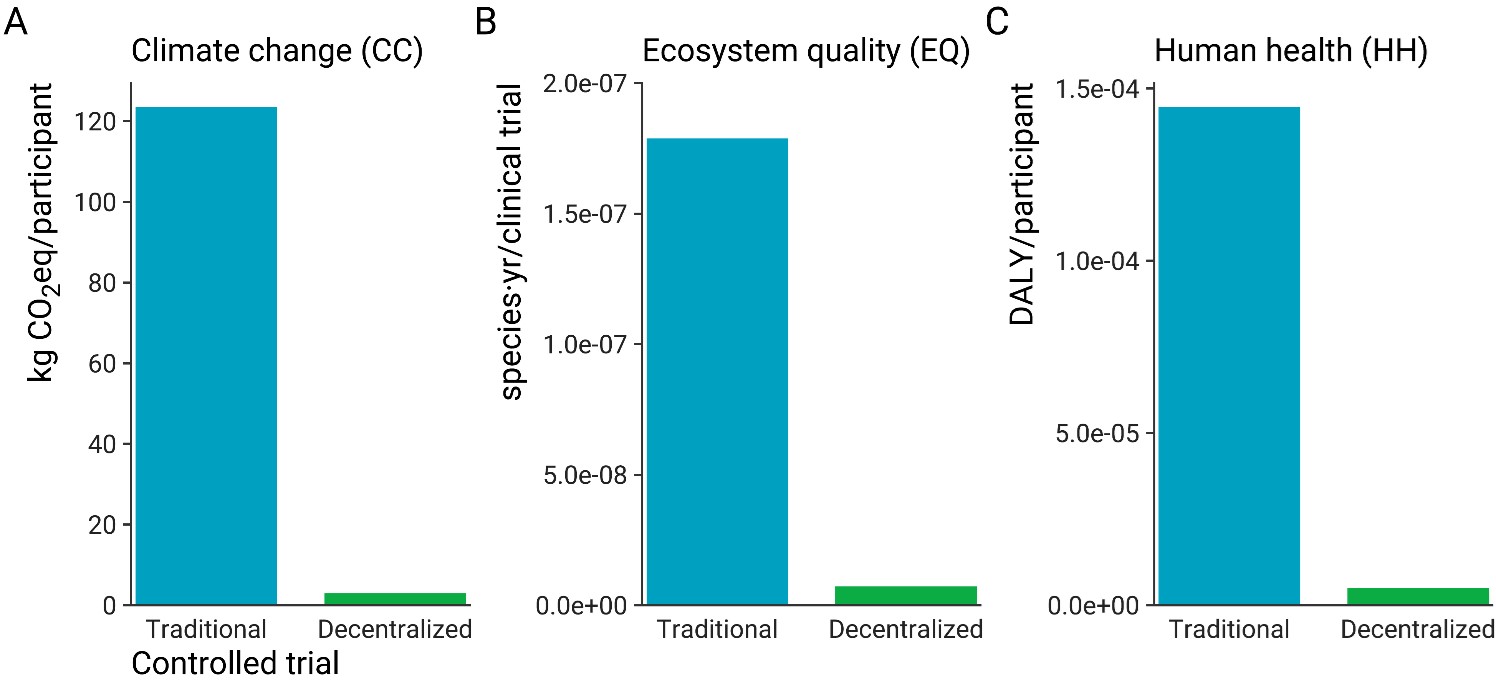

Supplement: Supplementary file 2 [file Image_2.jpg]

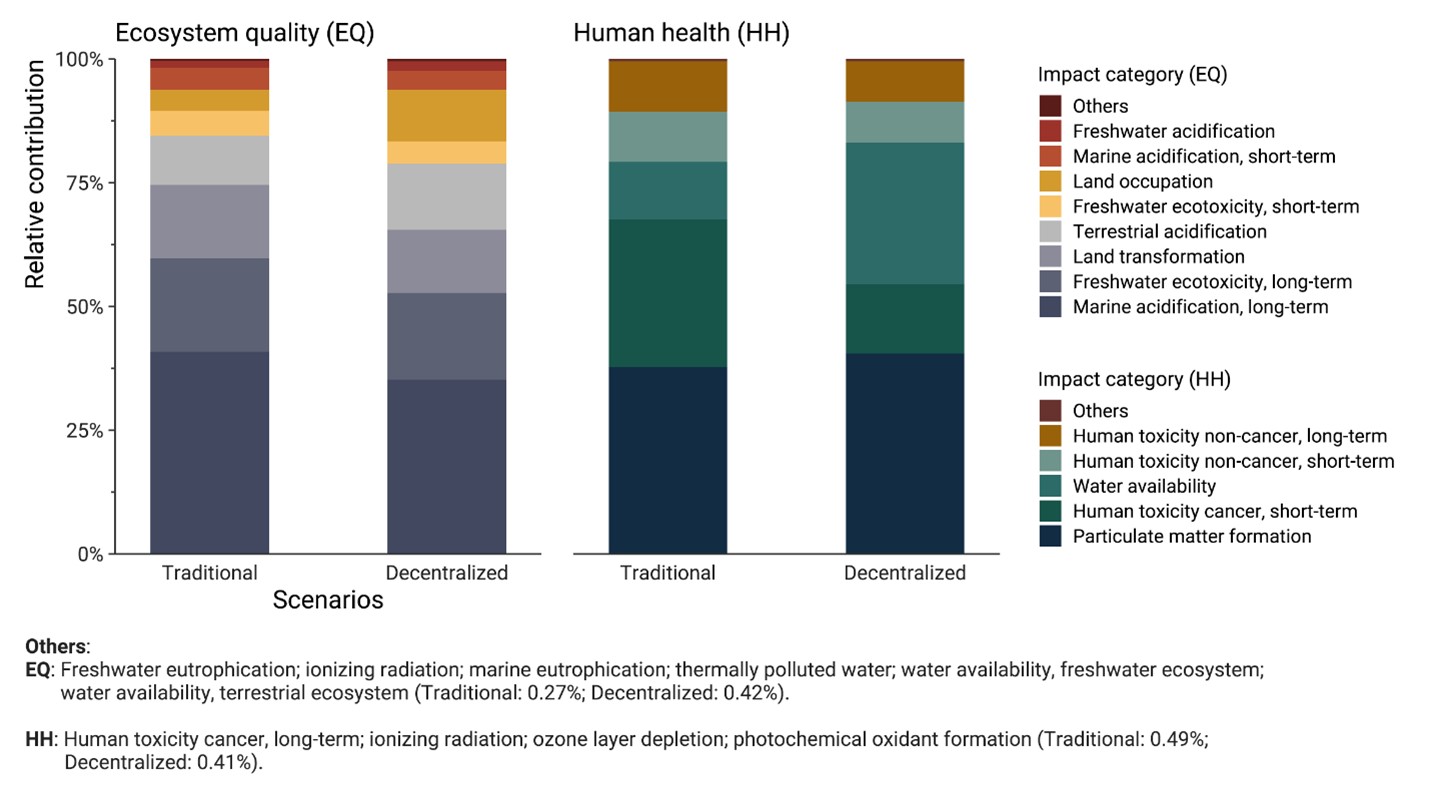

Supplement: Supplementary file 3 [file Image_3.jpg]
